# Identification of disease-related aberrantly spliced transcripts in myeloma and strategies to target these alterations by RNA-based therapeutics

**DOI:** 10.1038/s41408-023-00791-0

**Published:** 2023-02-03

**Authors:** Daisuke Ogiya, Zuzana Chyra, Sigitas J. Verselis, Morgan O’Keefe, Jacquelyn Cobb, Ivane Abiatari, Srikanth Talluri, Anjana Anilkumar Sithara, Teru Hideshima, Michael P. Chu, Roman Hájek, David M. Dorfman, Linda M. Pilarski, Kenneth C. Anderson, Sophia Adamia

**Affiliations:** 1 Department of Hematology and Oncology, Tokai University School of Medicine, Isehara, Japan; 2 Department of Hemato-oncology, University Hospital Ostrava, Ostrava, Czech Republic; 3 Department of Hemato-oncology, University of Ostrava, Ostrava, Czech Republic; 4 Molecular Diagnostic Laboratory, Dana-Farber Cancer Institute, Boston, MA USA; 5 Jerome Lipper Multiple Myeloma Disease Center, Dana-Farber Cancer Institute, Harvard Medical School, Boston, MA USA; 6 Institute of Medical and Public Health Research, School of Medicine, Ilia State University, Tbilisi, Georgia; 7 Veterans Administration Boston Healthcare System, West Roxbury, MA USA; 8 Department of Medicine, Department of Oncology, University of Alberta, Edmonton, AB Canada; 9 Department of Pathology, Brigham and Women’s Hospital, Harvard Medical School, Boston, MA USA; 10 Beth Israel Deaconess Medical Center, Harvard Medical School, Boston, MA USA

**Keywords:** Myeloma, Cancer

## Abstract

Novel drug discoveries have shifted the treatment paradigms of most hematological malignancies, including multiple myeloma (MM). However, this plasma cell malignancy remains incurable, and novel therapies are therefore urgently needed. Whole-genome transcriptome analyses in a large cohort of MM patients demonstrated that alterations in pre-mRNA splicing (AS) are frequent in MM. This manuscript describes approaches to identify disease-specific alterations in MM and proposes RNA-based therapeutic strategies to eradicate such alterations. As a “proof of concept”, we examined the causes of aberrant HMMR (Hyaluronan-mediated motility receptor) splicing in MM. We identified clusters of single nucleotide variations (SNVs) in the HMMR transcript where the altered splicing took place. Using bioinformatics tools, we predicted SNVs and splicing factors that potentially contribute to aberrant HMMR splicing. Based on bioinformatic analyses and validation studies, we provided the rationale for RNA-based therapeutic strategies to selectively inhibit altered HMMR splicing in MM. Since splicing is a hallmark of many cancers, strategies described herein for target identification and the design of RNA-based therapeutics that inhibit gene splicing can be applied not only to other genes in MM but also more broadly to other hematological malignancies and solid tumors as well.

## Introduction

Ongoing large-scale genomic analyses of samples from MM patients have identified previously unknown, high-frequency genetic variations (GVs), some of which are important “drivers” of the disease, while others are simply “passenger” alterations [1–3]. Whole-genome transcriptome analyses in a large cohort of MM patients demonstrated that epigenetic changes such as alterations in pre-mRNA splicing (AS) are frequent in MM [4]. Pre-mRNA splicing is executed in the nucleus by large macromolecular complexes called spliceosomes. The pre-mRNA sequence elements that determine splicing specificity are classical splice sites such as 5’ (donor) and 3’ (acceptor) splice sites, a splicing branch point (BP), and polypyrimidine tracts (PPTs) of splicing [5–7]. The efficiency of this process is subject to control by cis-splicing elements, such as exonic and intronic splicing enhancers (ESE, ISE), as well as exonic and intronic splicing suppressors (ESS, ISS) [5]. GVs detected in splicing regulatory elements (SREs), which include classical and cis-splicing elements, have led to the discovery of aberrant splicing in many disease-related genes. Importantly, these splicing alterations create functionally significant disease biomarkers and drug targets [8–10].

mRNA transcripts that bridge transcription and translation, two major cell mechanisms, are ideal targets for developing selective therapeutic approaches. These approaches, which are a type of precision medicine commonly referred to as “RNA therapeutics,” allow for gene-selective specificity. They can target disease-causing GVs, modulate splicing alterations, or control epigenetic modulators (miRNA, snRNA, and lncRNA) of the cancer genome. Moreover, RNA therapies can also target alterations that are “undruggable” by small molecules or proteins. RNA therapeutic agents, used alone or in combination with conventional therapies, include antisense oligonucleotides (ASOs), aptamers, siRNAs, and more recently, miRNAs and synthetic mRNAs [11–14]. Clinical use of ASO-based therapies has been hindered due to ASO toxicity and limitations associated with their uptake by tumor cells. However, advances in medicinal chemistry now allow for RNA-based therapy that is more potent and less immunogenic [15–17]. Some RNA-based drugs have been approved by the FDA, and others are in pre-clinical development and/or in clinical trials [18–23].

In MM, aberrantly spliced genes including *XPB1, MMSET, CD44, HAS1, HMMR*, and *Gal-8* regulate cell proliferation and apoptosis [24–30]. Moreover, overexpression of selective splice variants of these transcripts in MM clones correlates with inferior patient survival [24, 25, 27, 28]. In this study, we have developed strategies to identify aberrantly spliced transcripts in MM, define their role in disease pathogenesis, and identify potential targets for RNA-based therapeutics. As a “proof of concept,” we examined HMMR splicing in MM and identified the most recurrent spliced transcripts of this gene. We then used SNPs as a tool to predict vulnerable regions of the HMMR transcript that are subjected to aberrant splicing and showed that targeting those vulnerable sequences by ASOs may to abrogate aberrant splicing products.

Based on bioinformatic analyses and validation studies, we provide the pre-clinical rationale for RNA-based therapeutic strategies that selectively inhibit the production of HMMR splice variant transcripts in MM. Importantly, since gene splicing is a hallmark of many cancers, our paradigm for target identification and RNA-based therapeutics to inhibit gene splicing can be applied not only to other genes in MM, but also more broadly to other hematological malignancies and solid tumors as well.

## Materials and methods

### Tissue and cell preparation

Diagnostic samples from 48 MM patients were obtained after IRB-approved informed consent. Patient samples were de-identified before arrival in the research laboratory. Sample preparation and RNA isolation were done as previously reported [30, 31]. Total RNA isolated from the healthy donor bone marrow (BM) aspirates were used as a control in gene profiling experiments. Human cell lines 293T and NCI-H929 obtained from the American Type Culture Collection and maintained according to the manufacturer’s recommendations.

### HMMR expression and splicing pattern analyses

cDNA for the RT-PCR and PCR reactions was prepared, as described previously [28]. The HMMR primer sequences are 5’-AGTGCCAGTCACCTTCAGTTTCT-3’ and 3’-ATTTAGCCTTGCTTCCATCTTTT-5’. Amplicons were detected using gel electrophoresis or capillary electrophoresis and DNA fragment analysis on the ABI 31/30XL DNA genetic analyzer (Thermo Fisher Scientific, Waltham, MA). Capillary electrophoresis and DNA fragment analyses were done as described previously [28].

### Transient transfection of the splicing cassettes

Transient transfections studies were carried out on 293T and NCI-H929 cell lines. We used lipofectamine 2000 for 293T transfection experiments, while NCI-H929 was transfected using the Neon electroporation system (Invitrogen^TM^, Carlsbad, CA), according to the manufacturer’s recommendations. The HMMR minigene that included introns 3 and 4 and an exon 4 was synthesized and subcloned into a minigene splicing reporter derived from the Fibrinogen Bβ minigene pT-Bβ-442 IVS7 + 1 G > T plasmid that was previously described [32]. Cells were transiently transfected with 1 μg of HMMR minigene construct. RNA isolated from the cells was subjected to RT-PCRs using HMMR primer pairs. The PCR amplicons were separated on a nondenaturing 2% agarose gel or detected by capillary electrophoresis and DNA fragment analysis.

### Western blotting

The NCI-H929 cells, transfected with or without PTBP1 and PTBP2, were lysed using RIPA buffer with protease inhibitors (Stem Cell Technologies, Vancouver, Canada) and transferred onto a nitrocellulose membrane (Millipore, Bedford, MA). Nonspecific binding was blocked by 5% BSA/0.1% Tween 20 PBS blocking buffer (#9997, Cell Signaling Technology, Danvers, MA). The membrane was incubated with anti-HMMR antibodies from Cell Signaling Technology (#87129).

### Antisense oligonucleotides (ASO)

Control ASOs or siRNA targeting HMMR-FL and HMMR-V3 were designed by us and synthesized at the Qiagene. Scrambled ASO and/or ASO targeting GAPDH were used as negative and positive controls, respectively. The NCI-H929 MM cells were transduced with ASOs at 100 nM final concentration, by gymnosis or electroporation using the Neon transfection system, according to the manufacturer’s recommendations (Life Technologies). The ASO/siRNA delivery efficacy and HMMR-FL/V3 knockdown were evaluated at transcript and protein levels by RT-PCR followed by capillary electrophoresis and DNA fragment analyses, and western blotting [28]. The ASOs functional effects were evaluated by apoptosis assay via Annexin-V staining according to the manufacturer’s instructions (Biolegend), and genomic instability was observed by Micronuclei assay. Micronuclei were quantified using a flow cytometry-based Micronucleus Assay kit (MicroFlow kit, Litron Laboratories), according to the manufacturer’s protocol and as previously described [33].

### Bioinformatic analysis

The classical and *cis*-splicing elements of the HMMR gene were assessed using web-based bioinformatics tools. Bioinformatic analyses were performed on wild-type (WT) or mutated (MT) exons [4] and introns [3, 4] of the HMMR gene using MaxEntscan, SpliceAid, and HSF (Human Splicing Finder) tools. The SpliceAid tool searches the sequence motifs of cis-splicing elements (ESE, ISE, ISE, ISS) that are recognized by SRE binding proteins and are derived from the pool of functional enhancer sequences tested experimentally in vivo and in vitro systems. In addition, cis-splicing elements, classical splicing branch points (BP), and polypyrimidine tracts (PPT) of WT and MT HMMR gene segments were mapped and evaluated using the HSF tool. The prediction analyses were carried out using default values, which were adjusted for background nucleotide composition.

## Results

### Identification of the most frequent and recurrent HMMR splicing events in MM

**Fig. 1:**
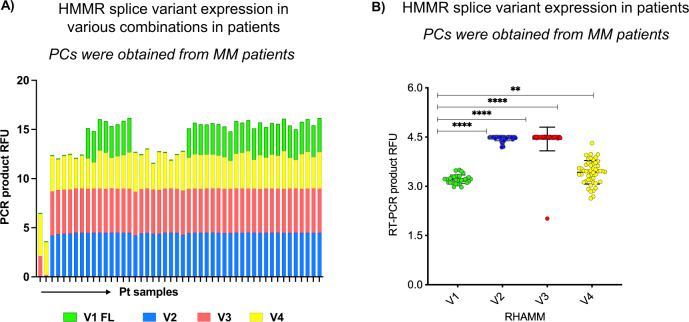
HMMR splice variant transcripts are frequently expressed in MM patients. **A**, **B** HMMR transcript expression was evaluated by RT-PCR followed by DNA fragment analyses and capillary electrophoresis. On the figures, the *y* axes display relative fluorescent units (RFU) of the PCR products. PCR product RFU = lg(RFU). RFU is a unit of measurement calculated relative to the size standards included in each reaction and compared to HMMR expression values detected in healthy donor BM PCs. In panel **A**, the *x* axes display patient samples, while in (**B**) the *x* axes display splice variants of HMMR. **A** displays splice variant co-expressions within individual patient samples (each bar represents one patient sample). **B** displays individual HMMR splice variant expression levels in MM patient samples.

We evaluated expression patterns of full-length HMMR (HMMR-FL) and four alternatively spliced transcripts: HHMR V1, V2, V3, and V4 (Supplementary Fig. 1A, B) in CD138 + plasma cells (PCs) obtained from bone marrow (BM) aspirates of MM patients and healthy donors (HD) (Fig. 1A, B) using RT-PCR and DNA fragment analysis followed by capillary electrophoresis. We detected ubiquitous expression of HMMR splice variants in MM-PCs in various combinations; HMMR-V2, V3, and V4 transcripts were significantly overexpressed in 97% of MM patients than HMMR-V1 and FL (66%) (*P* < 0.0001) (Fig. 1B). This analysis detected unbalanced overexpression of HMMR-V2 and V3 variants (Fig. 1B). In the majority of MM patients, the HMMR-V2/V1 and V3/V1 transcript expression ratios were > 2 fold (*P* < 0.001) high while, in HDs, the HMMR-V4/V1 transcript expression ratios ranged between 0.9 and 1.3-fold (Fig. 1A).

**Fig. 2:**
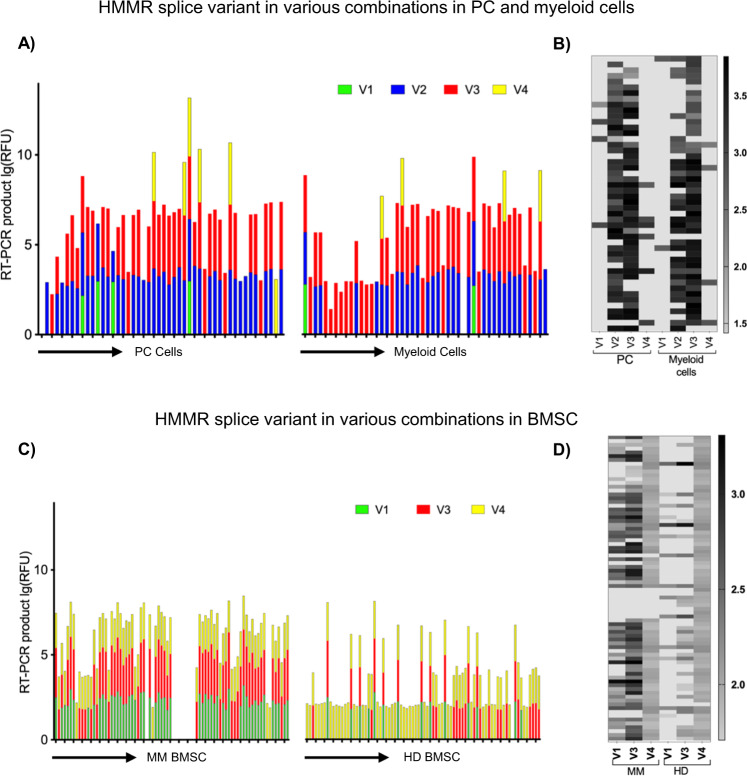
HMMR splice variant transcripts are frequently and recurrently expressed in different subpopulation of cells from MM patients. This figure displays HMMR splice variant expressions at a single-cell level. MM or HD cells were sorted by index sorting followed by RT-PCR DNA fragment analyses and capillary electrophoresis. **A**, **B** display HMMR splice variant expression at the single-cell level in MM-PCs and myeloid cells, while **C** and **D** display the expression of those variants in autologous MM BMSCs and HD BMSCs. **A**, **C** show HMMR splice variant co-expression levels. In these figures, the *x* axes display cells and the *y* axes relative fluorescent units (RFU) of the PCR products. PCR product RFU = lg(RFU). RFU is a unit of measurement calculated relative to the size standards included in each reaction. **B**, **D** The overall distribution of the HMMR splice variants in different subpopulations of MM and HD samples are presented as heatmaps. The color scale for expression values is shown on the right.

Considering that HMMR splice variants are expressed in tissue-type- and/or cell-type-specific manner, HMMR variant expression profiling was performed at a single-cell level in different subpopulations of BM cells in patients with relapsed refractory (RR) MM. Specifically, single-cell analyses were carried out in PCs, in BM-infiltrating myeloid cell populations, and in autologous BMSCs (Fig. 2 and Supplementary Fig. 2). The majority of PCs (70%) and myeloid cells (60%) expressed the HMMR-V3 variant in combination with HMMR-V2 transcripts, while HMMR-FL, -V1 and -V4 variants were expressed in only few PCs and myeloid cells (Fig. 2A, B). In addition, HMMR-V3 splice variant, which lacks a microtubule-binding domain and is associated with poor survival in patients with MM, was expressed alone in 34% of the myeloid cells and in 11% of the PCs in patients with RRMM (Fig. 2B) [27]. Furthermore, HMMR splice variant profiling at the single-cell level showed that HMMR-V3 was expressed in 64%, HMMR-V1 in 53%, and HMMR-V4 in 89% of MM BMSC, respectively, whereas HMMR-V2 was absent from these cells (Fig. 2C, D). Importantly, the HMMR-V3/V1 ratio was lower in MM BMSC (1.1-fold) than MM-PCs (>2.6-fold) (Fig. 2D). In contrast, the expression of these variants was significantly less in HD than MM BMSC (*P* < 0.0001; Fig. 2C, D). These studies showed distinct overexpression of HMMR-V3 splice variant by MM tumor cells (PCs). As previously described, this splice variant of HMMR is the result of exon 4 skipping (48 bp), which does not cause any frameshift and encodes functional protein [34].

### The impact of SNPs on HMMR gene splicing outcomes

Having shown the altered splicing pattern of HMMR in MM patient samples, we next examined potential causes for the exclusion of exon 4 in the HMMR pre-mRNA transcript. Exclusion of exon 4 leads to the synthesis and unbalanced expression of HMMR-V3 [35, 36]. It is well known that GVs can impair splicing regulatory elements (SREs) and consequently modulate the binding efficiency and distribution of splicing proteins (SRs or hnRNPs) on exons and introns, thereby altering splice site recognition and splicing pattern. Thus, we decided to use GVs as a tool to identify regions of the HMMR that are prone to splicing alterations.

We examined the incidence of GVs previously identified in the HMMR gene segment where the altered splicing took place. We also mapped GVs in the intronic regions flanking exon 4, as these regions overlap with its 3’ and 5’ splice sites, a BP, and the PPT of splicing (Supplementary Fig. 3). A total of 72 GVs were identified in HMMR exon 4 and flanking introns 3 and 4 reported in publicly available databases (ENSEMBL, NCBI) or in MM patients (COMPASS dataset, Supplementary Table 1). The majority (46 of 72, 74%) of GVs were mapped to the intronic regions of HMMR, while 16 of 72 (26%) were in the coding segment of exon 4 (Supplementary Table 1). These GVs include 35 intronic and 13 exonic SNPs (12 missense and 1 synonymous), 6 intronic and 1 exonic splice site SNPs, 5 intronic deletions, and 2 exonic somatic SNVs.

To evaluate the putative effects of GVs on HMMR splicing, we used bioinformatic tools to predict impaired splice site recognition by weakening either the intrinsic strength of the splice site (MaxEntScan) or its sequence neighborhood (HEXplorer). These analyses of HMMR intron 3 identified several hexamers: intronic silencers (ISSs) in the PPT of the splicing area of exon 4. This hexamer combination is disrupted by the SNP rs561052191 (∆HZEI = 113.36 HEXplorer score), which could decrease the U2AF65 core-splicing factor binding affinity to the PPT (Supplementary Fig. 3A, B). This prediction is supported by a slight decrease in the intrinsic strength of the splice acceptor upstream of the exon 4 site, as defined by the MaxEnt score (∆MaxEnt 6.71 > 5.62 (Table 1)). Using the MaxEnt tool, we also identified the SNP (T > A) rs369485598 (∆MaxEnt 6.71 > 2.44) as a splicing SNP, because it is located in the same intrinsic PPT site upstream of exon 4 (Table 1). These two SNPs therefore have a high potential for weakening PPT strength and thus could potentially alter splice acceptor recognition upstream of exon 4.

**Table 1:** The impact of SNPs on the intrinsic strength of WT and MT HMMR exon 4 splice donor and acceptor sites.

Sequence	HBS	GVS
CAGGTAAGTAT		U1snRNA
gAGGTAAGcAg	17.3	WT
gAaGTAAGcAg	12.6	rs1175449655
gAGGTtAGcAg	13.4	rs988517256
gAGGTAAGTAg	20.8	rs752321701
gAGGTAAGcAa	17.3	rs755740711
gAaGTtAGTAa	9	All SNPs

Sequence	MaxEnt 3’ss	GVS
TTTTTTTGGCTTTTAAACAGAAG	6.71	WT
TgTTTTTGGCTTTTAAACAGAAG	6.27	rs762058216
TTTTgTTGGCTTTTAAACAGAAG	5.62	rs561052191
TTTTTaTGGCTTTTAAACAGAAG	6.4	rs1256927848
TTTTTTaGGCTTTTAAACAGAAG	2.44	rs369485598
TTTTTTTGtCTTTTAAACAGAAG	8.45	rs202024983
TTTTTTTGGCTTTTAAgCAGAAG	3.91	rs1235517851
TTTTTTTGGCTTTTAAAtAGAAG	5.47	rs1202181775
TGTTGAAGTCTTTTAAGTAGAAG	−1.59	All SNPs

Furthermore, prediction studies identified the SNP rs1235517851 as a potential cause of HMMR-V3 splice variant expression (Supplementary Fig. 3A, C). This intronic SNP is located upstream of the AG dinucleotide of the splice acceptor (3’ site). Although SNP rs1235517851 does not interfere with the “CAG” intronic consensus sequence at the 3’ splice site, it weakens the intrinsic strength of the 3’splice acceptor site (ΔMaxEnt 6.76 > 3.96) (Table 1). It also creates an additional putative 3’ acceptor splice site (AG dinucleotide), due to its A > G nucleotide change. This alteration could lead to only modest binding of U2AF35 to the 3’ acceptor splice site in the HMMR at exon 4, which ultimately could compromise exon 4 recognition and consequently lead to the synthesis of HMMR-V3 variant transcripts. The creation of an additional 3’ splice site as a result of SNP rs1235517851 weakens the intrinsic strength of the constitutive 3’ acceptor splice site by changing the MaxEnt score from 6.76 to 3.96 (Table 1). Thus, these prediction studies may suggest an association between the SNP rs1235517851 and exon 4 exclusion from the HMMR pre-mRNA (Supplementary Fig. 3C).

GV mapping analyses in the HMMR gene identified a subset of SNPs located in 5’ splice donor (SD) sites which influence the recognition of these intrinsic SD sequences by the spliceosomal subunits, especially U1 snRNP. U1snRNA forms a duplex upon recognition of an 11-nucleotide SD site. Intrinsic SD strength is highly dependent on the base pairing complementarity of the nucleotides at this site. The recognition of an SD sequence can be affected by nucleotide changes. We next evaluated the effects of the SNPs overlapping with the 11 bp consensus sequence in the exon/intron 4 junction (SD site at exon 4) of the HMMR transcript.

Among the analyzed SNPs, rs1175449655 and rs988517256 are located at −1 (the last exonic nucleotide on exon 4) and + 3 (the 3rd intronic nucleotide on intron 4) positions on the SD site, respectively. These most conserved (>75% in humans) nucleotides form strong bonds with U1 snRNPs, which are core subunits of spliceosomes. In silico prediction analyses showed that these SNPs decrease the SD site strength, as measured by the decreased HBS (H-bond score) in SNP rs1175449655 (HBS = 12.60) and SNP rs988517256 (HBS = 13.40) as compared to SD sequence (HBS = 17.30) (Supplementary Fig. 3D and Table 1). These alterations would compromise snRNA binding to the intrinsic SD site and facilitate HMMR exon 4 skipping, resulting in an elevated V3/V1 ratio. The SNP rs755740711, located at the + 8 positions of the SD site, did not change the HBS as compared to the HBS of the consensus SD sequence (Table 1). Therefore, we did not test this mutation in validation studies.

**Fig. 3:**
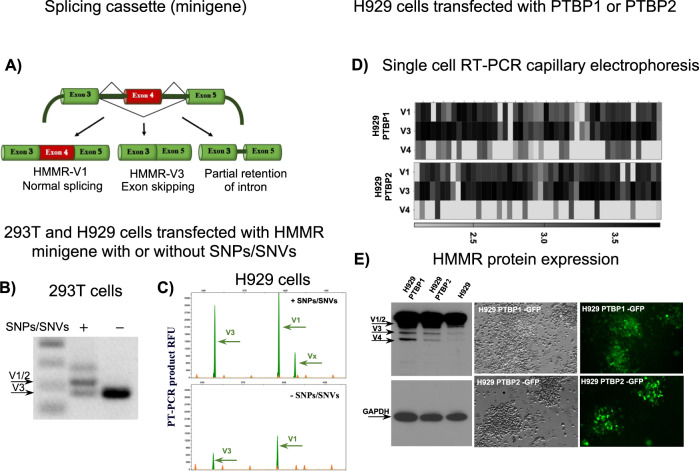
Validation studies effects of SNPs and splicing factor deregulation on HMMR splicing. **A** Schematic representation of HMMR minigene splicing cassette. HMMR exons 3–5 are represented in green and red boxes; dark green lines represent the shortened introns; expected HMMR splice variant transcripts are shown on the figure. **B** Gel electrophoresis result of the functional splicing assay of the wild-type and mutated HMMR minigene in 293T cells. **C** Capillary electrophoresis result of the functional assay of the wild-type and mutated HMMR minigene in NCI-H929 cells. The full-length and splice variant transcript are shown as green peaks. The Genescan Liz-1000 size standard is shown as orange peaks. Fragment sizes and relative fluorescent units (RFU; RFU = lg(RFU)) are indicated on the *x*- and *y* axes, respectively. **D**, **E** HMMR splice variant transcript (**D**) and protein (**E**) expression in the NCI-H929 cells stably transfected with PTBP1- and PTBP2-GFP constructs. **D** shows single-cell RT-PCR capillary electrophoresis results; HMMR splice variant distributions in MM and HD BMSC samples are presented as heatmaps. The color scale for expression values is shown. **E** shows western blotting and DIC and fluorescence images of NCI-H929 cells overexpressing PTBP1-GFP and PTBP2-GFP. Protein lysates of transfected and parental cells were separated on SDS-PAGE, blotted onto nitrocellulose, and probed with anti-HMMR antibodies. Arrows identifying HMMR-V1/2, V3, and V4 bands are shown.

We next used an ex vivo splicing assay to evaluate the functional effects of SNPs/SNVs on HMMR splicing. We generated an HMMR splicing cassette that included minigenes (exons and introns) of HMMR, with and without GV predicted to have effects on HMMR splicing (Fig. 3A). In validation studies, we included the SNPs mapped within either of the surrounding splice sites in the coding region of HMMR, as well as SNP rs767100503, which is located in the middle of exon 4. This silent T > C SNP does not cause amino acid changes. However, it is capable of altering putative downstream splicing regulatory elements and compromising SRp40 binding to HMMR, as predicted by the human ESE finder (Supplementary Figs. 3E and 4). SRp40 interacts with the heterodimeric auxiliary factor U2AF65, which is an essential subunit of the splicing factor U2AF.

293T and NCI-H929 MM cell lines were transfected with HMMR minigenes, with or without predicted SNPs/SNVs (Fig. 3A). RT-PCR analyses with reporter-specific primers (including the minigene) were performed on total RNA obtained from the minigene-transfected cells. These analyses showed that selective recurrent SNP clusters detected in the HMMR gene might contribute to aberrant HMMR pre-mRNA splicing, leading to an increased HMMR-V3 transcript expression (Fig. 3A–C).

We tested the effects of PTBP1/2 (polypyrimidine tract binding protein) deregulation on HMMR splicing, since some of the GVs on the HMMR modulate canonical binding sites of these splicing factors in the PPT of splicing. Additionally, it is known that PTBPs are involved in exon exclusion. We overexpressed PTBP1/2 in NCI-H929 MM cells and evaluated the HMMR splicing pattern in transfected cells at a single-cell level using RT-PCR DNA fragment analyses and capillary electrophoresis (Fig. 3D). These analyses identified significantly increased (*P* < 0.0001) expression of V1, V3, and V4 variants in PTBP1- or PTBP2-transfected compared to wild-type cells. The single-cell analyses identified overexpression of the V3 splice variant in 46% of NCI-H929 cells expressing PTBP1 and in 47% of NCI-H929 cells expressing PTBP2, exclusively in combination with the V1 variant. These single-cell analyses showed that overexpression of PTBP1/2 increased (2.5-fold) the V3/V1 ratio in in NCI-H929 MM cells (*P* < 0.0001) (Fig. 3E).

### Inhibiting HMMR-FL and splice variants by ASOs and evaluation their functional effects

**Fig. 4:**
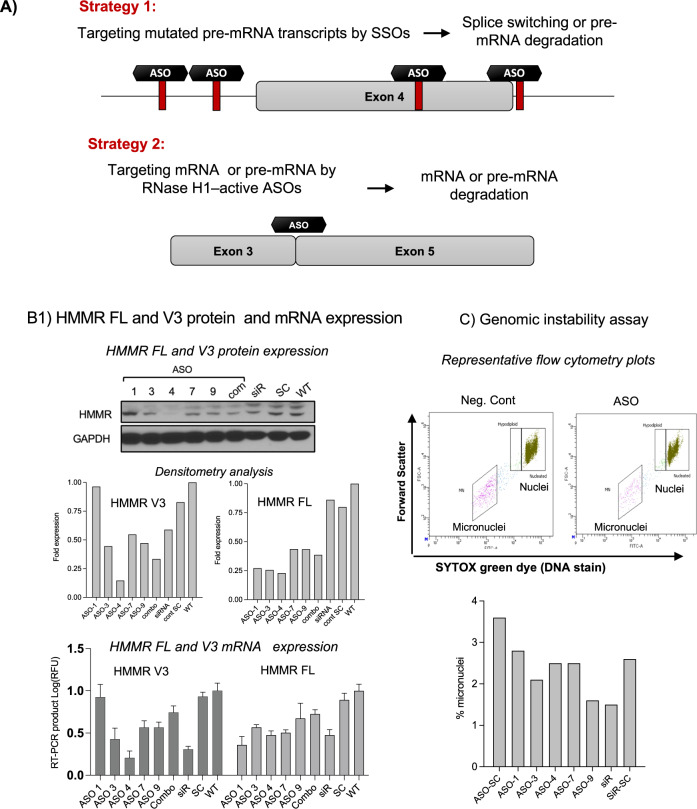
Targeting strategies using an antisense oligonucleotide (ASO)-based approach. **A** shows a schematic diagram of two strategies describing therapeutic approaches to modulate HMMR splicing in MM patients. Strategy 1: SNPs that induce aberrant HMMR splicing can be used as targets for splice-switching antisense oligonucleotides (SSOs) by degrading pre-mRNA transcripts without recruiting RNase H1. Strategy 2: use of RNase H1-active ASOs. DNA-like antisense oligonucleotides, which span exon junctions of mis-spliced mRNA, interact with their target mRNA in the cytoplasm or pre-mRNA in the nucleus of a cell, recruit RNase H1, and mediate the selective degradation of the target RNA molecule within the DNA:RNA duplex. **B** is a summary of the experiments that demonstrate HMMR transcript and protein inhibitions by ASOs. HMMR-Fl and HMMR-V3 knockdown efficiency was evaluated at protein and mRNA levels. After gymnosis, cell lysates were collected from each sample for western blotting analyses and total RNA was isolated for RT-PCR DNA fragment analyses and capillary electrophoresis. **B** The results are presented as RFU (relative fluorescent units) to GAPDH RFU. Protein lysates were subjected to immunoblotting using anti-HMMR antibodies; GAPDH served as a loading control. Bar graphs show a densitometric analysis of the HMMR-FL and V3 protein bands measured by ImageJ software. Fold expressions on the *Y* axis shows the protein expression level compared to loading controls. **C** is a summary of the genomic instability assay. After 48 h of gymnosis, cells were collected and viable cells were separated using Ficoll-Paque PLUS. Micronuclei were quantified by flow cytometry staining using In Vitro MicroFlow Kit (Litron Labs). Images of micronuclei (flow cytometry plots) and bar graphs showing the percentage of micronuclei are presented.

There are many ways to target HMMR gene splicing. Considering toxicity, efficacy, and tissue uptake of RNA, we propose two viable strategies (Fig. 4A). One of viable strategy is to target and abrogate the unbalanced expression of HMMR-V3 in MM cells by designing antisense oligonucleotides (ASO) against segments of HMMR pre-mRNA, specifically across the weak polypyrimidine tracts of splicing that contribute to HMMR exon 4 skipping. We designed ASO specifically targeting coding and non-coding regions of HMMR that contribute to altered HMMR gene splicing as we predicted by bioinformatic analyses. ASOs or siRNA targeting HMMR were delivered into the NCI-H929 MM cell line. Cells were harvested 48 h after delivery. The ASO delivery efficiency raged from 80 to 97% (Supplementary Fig. 4). The HMMR transcript and protein inhibition by ASOs were evaluated at the transcript and protein levels (Fig. 4B). These analyses identified that ASOs mapped to the classical splicing elements upstream of exon 4 lead to total inhibition of HMMR-FL and splice variant V3 expression (Fig. 4B). We evaluated the functional effects of the ASO treatment on the NCI-H929 cells by monitoring micronuclei formation as a marker for genomic instability (Fig. 4C). Of all the ASOs tested, SSO showed >50% reduction in micronuclei content, suggesting reduced genomic DNA damage in myeloma cells. Similarly, >40% reduction in micronuclei content was detected after HMMR knockdown in NCI-H929 cells by siRNA (Fig. 4C).

## Discussion

Despite the development of novel targeted therapies in MM, ongoing DNA damage and clonal evolution underlie disease relapse in most patients. To address this issue, as a “proof of concept”, we examined the causes of aberrant HMMR splicing in MM. Based on GVs, we identified the most vulnerable regions on the HMMR gene subjected to altered splicing. Then, we validated novel RNA therapeutics targeting these regions. Targeting HMMR has been previously evaluated with HMMR peptide vaccination in MM and other malignancies [37–39]. Although HMMR peptide-specific immune responses were induced, clinical outcomes were mixed [40–42]. This lack of clinical efficacy may be due to variable HMMR expression on subpopulations of tumor and normal cells. Our study identified an HMMR-V3 splice variant to be significantly overexpressed in MM patients. Furthermore, by profiling HMMR splice variant expressions at a single-cell level in different subpopulations of MM cells, we identified HMMR-V3 to be significantly and recurrently expressed in both PCs and BMSCs in MM patients.

Selectively targeting splice variant proteins including the HMMR-V3 for immunotherapy can be challenging. The identification of specific immunogenic epitopes in splice variant proteins is difficult because they differ from the original protein or other splice variants by a few amino acids. For example, the HMMR-V3 protein different from other splice variants by 16 amino acids. This problem can be solved by targeting HMMR-V3 transcripts (mRNA) rather than the protein. Because the activity of RNA-based drugs relies on the base pairing of specific ASOs with their mRNA targets, there is a possibility of obtaining higher target specificity with this approach. RNA drugs can differentiate between target sequences that differ by a single nucleotide, and thus can identify them selectively [13, 14, 43]. These characteristics make ASOs a suitable therapeutic strategy to inhibit undruggable targets.

To target and “manipulate” HMMR splicing process by RNA-based agents, we used already-reported SNPs and GVs to predict vulnerable regions of the HMMR transcript that are subjected to aberrant splicing. While some genetic variations disrupt intrinsic splice sites, others create de novo splice sites or activate cryptic ones. Additionally, SNPs/GVs can modify SREs and alter the recruitment of splicing factors or compromise spliceosome assembly, consequently modulating splicing reactions [44]. We mapped a total of 72 SNPs/GVs reported in publicly available databases in the proximity of or directly located in HMMR exon 4, where altered splicing took place. We found that the majority (72%) of the SNPs/GVs were mapped in the intronic region of HMMR exon 4, and 28% were mapped in the coding region.

**Fig. 5:**
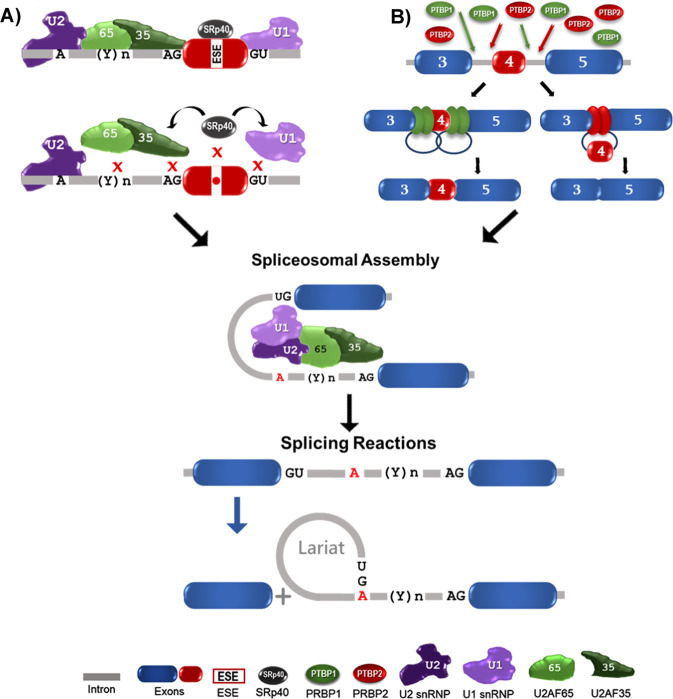
Model: effects of SNPs and splice factor deregulation on HMMR splicing. **A** demonstrates the effects of SNPs, while **B** shows the effects of PTBP1/2 overexpression on HMMR splicing. These alterations compromise complex A formation and downstream lead to alterations in the first etherification reaction. In the figure, “A” and “(Y)n” represent splicing branch point (BP) and polypyrimidine tract (PPT) of splicing, respectively.

Bioinformatic analyses of SNPs/GVs in the vicinity of HMMR exon 4 identified SNPs/GVs that affect splicing transesterification reactions, compromising the first steps of this mechanism. These SNPs/GVs are located within the pre-spliceosome, where assembly takes place (Fig. 5). In this assembly, U1 snRNPs and U2 snRNPs recognize 5′ splice sites and branch points of splicing, respectively, while U2AF35/AF65 bind to the 3’ site and the polypyrimidine tracts of splicing. When these proteins are assembled, the first transesterification reaction takes place (Fig. 5). SNPs rs561052191 and rs369485598 alter the U2AF65 binding site, while SNP rs1235517851 weakens the 3’ splice site and compromises the binding affinity of U2AF35. In addition, this SNP activates a cryptic 3’ splice site in exon 4, and potentially disrupts U2AF35/AF65 complex formation (Fig. 5). Furthermore, SNPs rs1175449655 and rs988517256, located in the 5’ site, have the ability to change the U1 snRNP binding affinity. SNP rs767100503, located in the coding region of HMMR exon 4, does not map to classical splicing sites (Fig. 5). This SNP might not affect HMMR exon 4 splicing; however, bioinformatic analyses indicate that this SNP alters exonic splicing enhancer (ESE) sites and compromises SRp40 binding to HMMR exon 4 (Supplementary Fig. 5). SRp40 interacts with U2AF65 and could modulate the first transesterification reaction of splicing (Fig. 5). Bioinformatic findings were validated in an in ex vivo splicing assay, which supports the idea that the predicted SNPs contribute to the exon 4 splicing of HMMR pre-RNA. Although the incidence of HMMR SNPs in MM patients is low, and their effects on HMMR splicing can be minimal, our bioinformatics analyses of HMMR exon 4 splicing in MM identified splicing elements in HMMR that represent ideal targets for RNA-based therapy. Targeting these elements by ASO demonstrated inhibition of HMMR-FL and splice variant V3 at mRNA and transcript levels and decreased micronuclei content, suggesting reduced genomic DNA damage in myeloma cells.

The polypyrimidine tract in intron 3 of HMMR has a high probability of accumulating PTBP proteins, regardless of the incidence of SNPs in the vicinity of classical splice sites in exon 4. Moreover, this region of HMMR is enriched with binding sequence motifs for PTBPs, which play a critical role in exon skipping [45–47]. Importantly, the upregulation of PTBPs is associated with malignant transformation in solid tumors, and we have observed progressive overexpression of PTBP1/2 in MM patients and its association with disease progression [47–51]. In this study, HMMR splice variant profiling at a single-cell level showed an increased V3/V1 transcript ratio associated with PTBP1/2 overexpression. This finding suggests that the unbalanced expression of HMMR-V3 results from the upregulation of PTBP1/2 in PCs and myeloid cells obtained from MM patients with relapsed refractory disease. Our test of HMMR splice variant expression at the protein level further suggests that the HMMR-V3 protein is upregulated in cells overexpressing PTBP1/2. Of note, our evaluation was limited to determining an increase in the V3/V1 ratio after PTBP1/2 overexpression at the protein level, since the anti-HMMR antibody cannot distinguish all HMMR splice variants. HMMR-V1 and HMMR-V2 differ by a single amino acid, which is difficult to distinguish by western blotting. Taken together, bioinformatic and validation studies of the HMMR gene have enabled us to identify specific sites on the gene that can be targeted by RNA therapeutic approaches. Even in patients where the mentioned SNPs are not detectable, this approach still identifies vulnerable regions of the HMMR transcript that can be subjected to aberrant splicing because there is a higher probability of the “leakiness” of these sequences and the binding efficacy of the splicing modulators can be compromised.

There are many ways to target HMMR gene splicing, in this manuscript we proposed two strategies to correct this alteration (Fig. 4). The first viable strategy to target HMMR gene splicing is to abrogate the unbalanced expression of HMMR-V3 in MM cells by designing ASOs against segments of HMMR pre-mRNA that include SNPs in the weak polypyrimidine tracts of splicing that contribute to HMMR exon 4 skipping. These ASOs are referred to as splice-switching oligos (SSO). They bind to a target sequence based on complementary base pairing and create a steric block to SR binding proteins, including the binding of PTBP1/2 to HMMR pre-mRNA. This leads to the activation of cryptic PPTs of splicing in HMMR intron 3. The SSOs are chemically modified so that RNase H1, an RNA-cleaving enzyme, is not recruited to degrade the HMMR pre-mRNA-SSO complex. Using similar strategies, two RNA-based drugs Eteplirsen and Nusinersen have recently been approved by the U.S. Food and Drug Administration (FDA) for Duchenne muscular dystrophy (DMD) and spinal muscular atrophy (SMA) patients [18, 21, 52–54]. Since both of these drugs are SSOs, they use a similar mechanism of action to abrogate altered splicing, which we describe here for HMMR in MM.

Another strategy to target HMMR gene splicing is to degrade HMMR-V3 mRNA using RNase H1-active ASOs (Fig. 4). A single‐stranded DNA‐like oligonucleotide ASO forms a DNA/RNA duplex when bound to its complementary target site, creating a substrate for RNase H1 and leading to the selective cleavage of the targeted HMMR transcript. After cleavage, the ASO is released to interact with another target RNA. RNase H1-active ASOs can therefore target the coding region of HMMR and modulate several splice variants of HMMR simultaneously. A similar strategy has been used clinically to treat homozygous hypercholesterolemia using Mipomersen and Inclisiran [19, 55], which are RNase H1-active ASOs that induce the degradation of mRNAs encoding apolipoprotein B and proprotein convertase subtilisin–kexin type 9 (PCSK9), respectively [56, 57]. These drugs use mRNA cleavage mechanisms to modify cholesterol disposition in patients with hypercholesterolemia. Another recent example is Inotersen, an RNase H1-active ASO, which targets and degrades both the splice variant and the full-length transcript of transthyretin (TTR) mRNA [58, 59]. This drug was approved for the treatment of hereditary transthyretin amyloidosis (hATTR) patients [59]. Alternatively, HMMR can be targeted using lncRNA HMMR-AS1 as described in solid tumors [60, 61]. Targeting this lncRNA instead of the HMMR splice variant would be beneficial if the ASO design against the splice variant failed. In our studies, we identified at least two ASOs that specifically downregulate either HMMR-V3 only or both HMMR-FL and HMMR-V3 transcripts. Thus, the paradigm for target identification and RNA-based therapeutics to inhibit gene splicing described here for HMMR as a proof of concept can be applied not only to other genes in MM, but also more broadly to other hematological malignancies and solid tumors as well.

## Supplementary information

Supp Figure 1

Supp Figure 2

Supp Figure 3

Supp Figure 4

Supp Figure 5

Supplementary Table1

Supp material

## Data Availability

All data analyzed during this study are included in this published article and its supplementary information files.
