# Supplementary material for: Identification of disease-related aberrantly spliced transcripts in myeloma and strategies to target these alterations by RNA-based therapeutics
Source: Blood Cancer J. 2023 Feb 3;13(1):23. doi: 10.1038/s41408-023-00791-0 (PMC9898564; doi:10.1038/s41408-023-00791-0)
Supplement: Supplementary file 3 — Supp Figure 3 [file 41408_2023_791_MOESM3_ESM.pptx]

## Slide 1
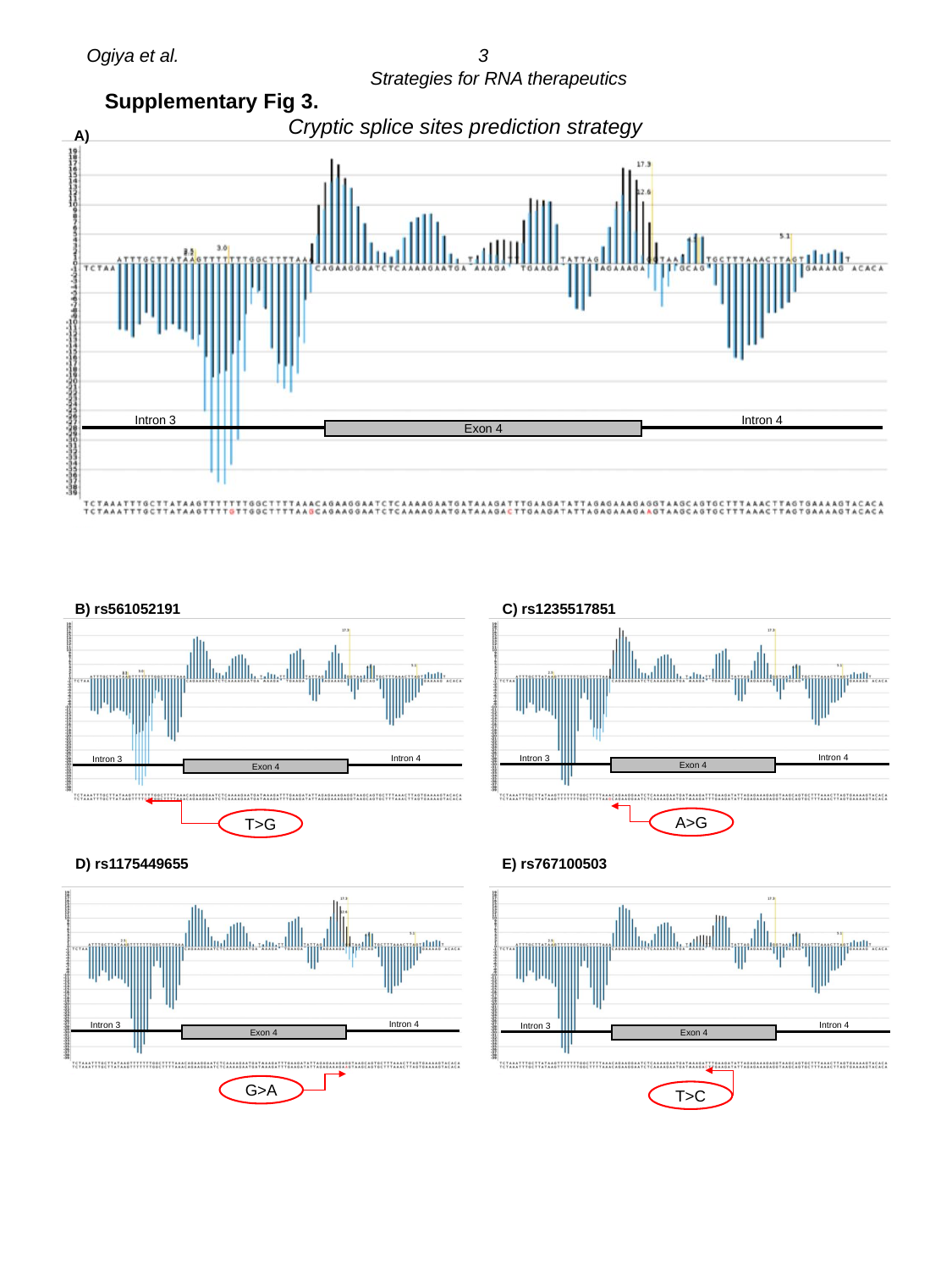

Ogiya et al. 3		Strategies for RNA therapeutics
Supplementary Fig 3.
Cryptic splice sites prediction strategy
A)
Intron 3
Intron 4
Exon 4
B) rs561052191
C) rs1235517851
Intron 4
Intron 3
Intron 4
Intron 3
Exon 4
Exon 4
A>G
T>G
D) rs1175449655
E) rs767100503
Intron 4
Intron 4
Intron 3
Intron 3
Exon 4
Exon 4
G>A
T>C
