# Supplementary figures and images for: Identification of disease-related aberrantly spliced transcripts in myeloma and strategies to target these alterations by RNA-based therapeutics
Source: Blood Cancer J. 2023 Feb 3;13(1):23. doi: 10.1038/s41408-023-00791-0 (PMC9898564; doi:10.1038/s41408-023-00791-0)

## Slide 1
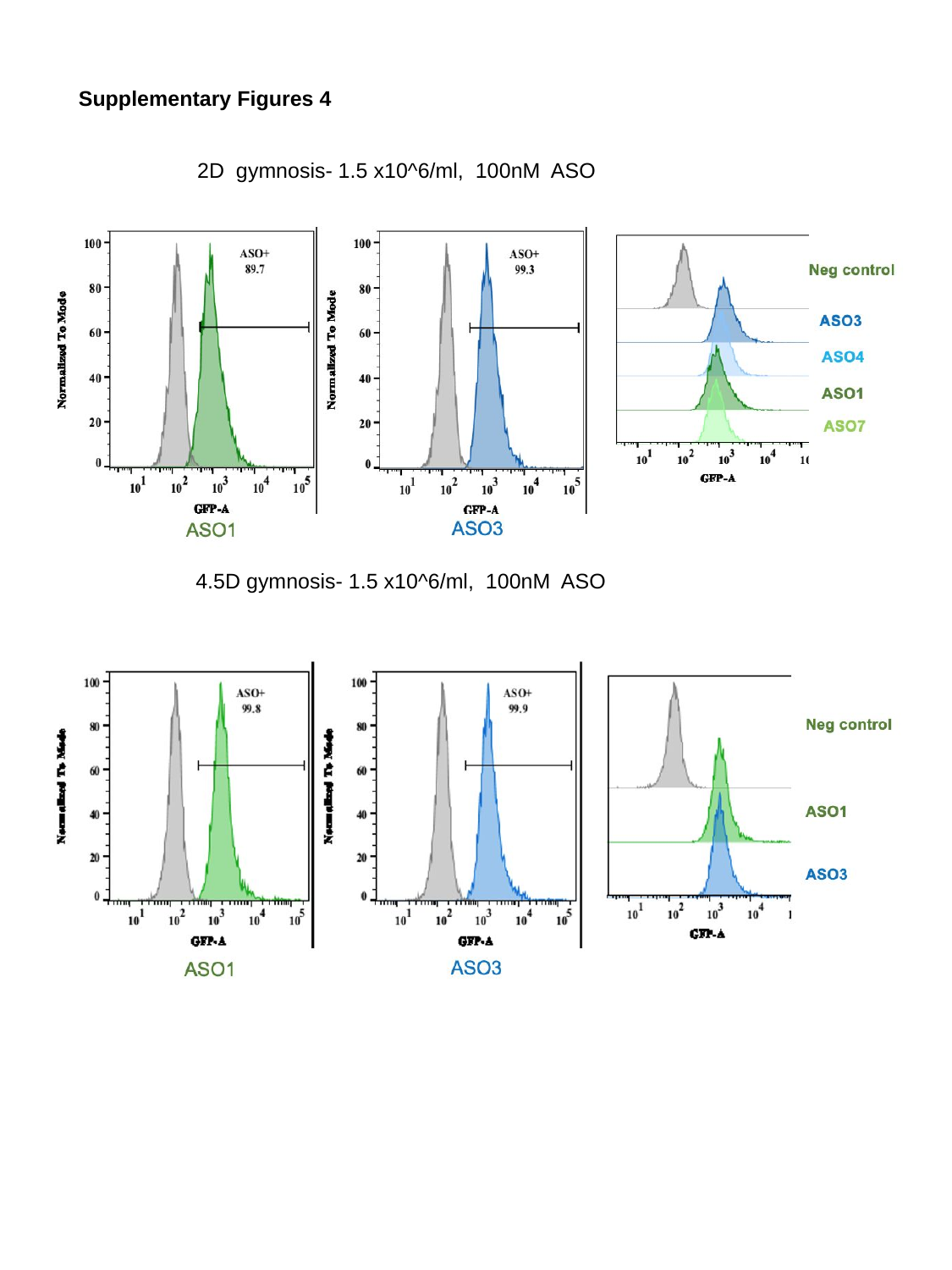

Supplementary Figures 4
2D gymnosis- 1.5 x10^6/ml, 100nM ASO
4.5D gymnosis- 1.5 x10^6/ml, 100nM ASO

Supplement: Supplementary file 4 — Supp Figure 4 [file 41408_2023_791_MOESM4_ESM.pptx]
