# Supplementary material for: Identification of disease-related aberrantly spliced transcripts in myeloma and strategies to target these alterations by RNA-based therapeutics
Source: Blood Cancer J. 2023 Feb 3;13(1):23. doi: 10.1038/s41408-023-00791-0 (PMC9898564; doi:10.1038/s41408-023-00791-0)
Supplement: Supplementary file 5 — Supp Figure 5 [file 41408_2023_791_MOESM5_ESM.pptx]

## Slide 1
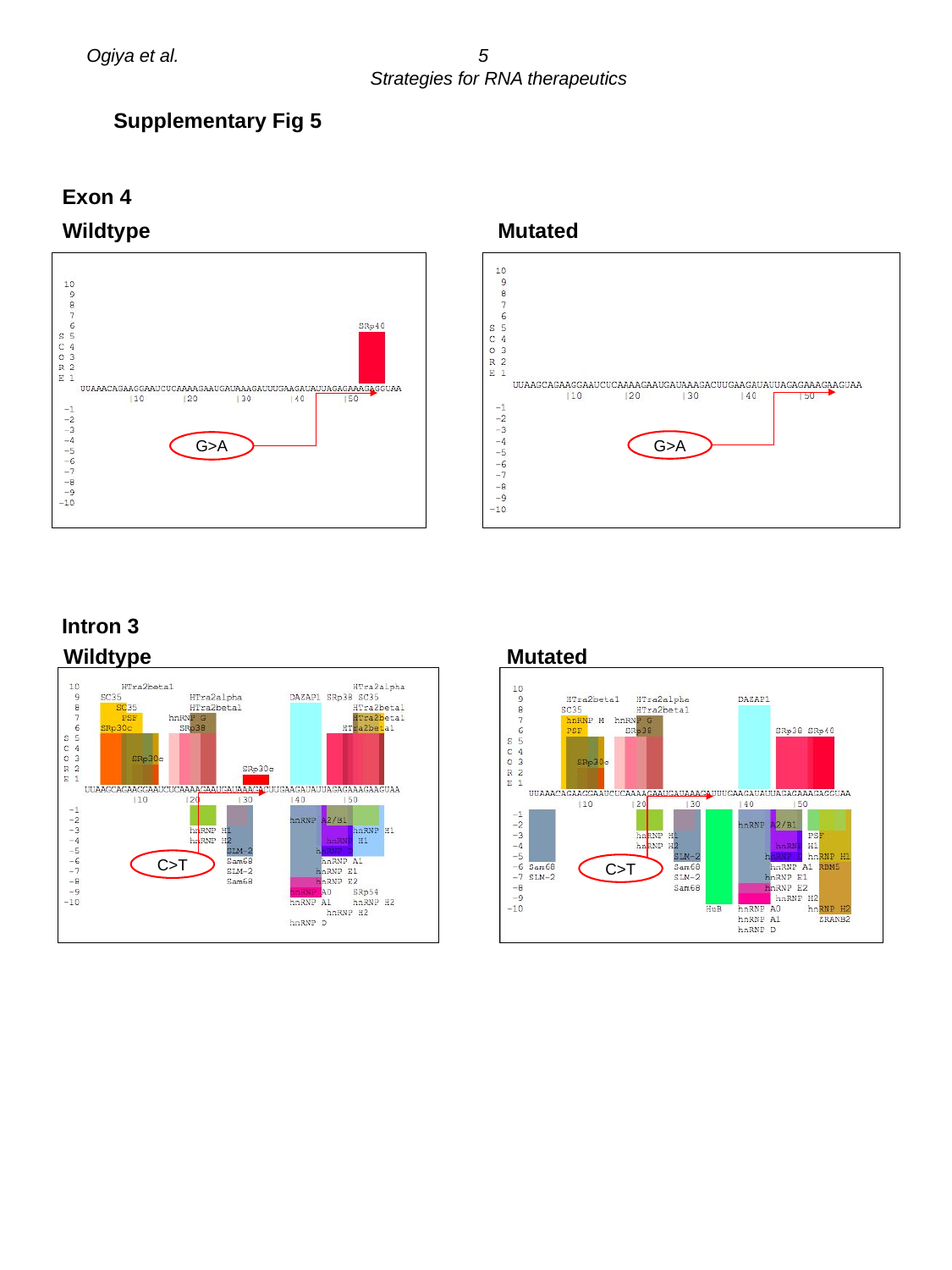

Ogiya et al. 5		Strategies for RNA therapeutics
Supplementary Fig 5
Exon 4
Wildtype
Mutated
G>A
G>A
Intron 3
Wildtype
Mutated
C>T
C>T
