# Supplementary material for: Identification of disease-related aberrantly spliced transcripts in myeloma and strategies to target these alterations by RNA-based therapeutics
Source: Blood Cancer J. 2023 Feb 3;13(1):23. doi: 10.1038/s41408-023-00791-0 (PMC9898564; doi:10.1038/s41408-023-00791-0)
Supplement: Supplementary file 7 — Supp material [file 41408_2023_791_MOESM7_ESM.docx]

Supplementary Figuer legends

Supplementary Fig 1. RHAMM splice variant transcripts are frequently expressed in MM patients

(A) Schematic diagram of the HMMR splice variant transcripts (left) and proteins (right). Four different isoforms of HMMR—V1, V2, V3, and V4—are produced due to alternative splicing; the V1 splice variant encodes the longest full length (FL) isoform; the V2 variant uses an alternate acceptor splice site (3' site) at one of the coding exons. The V1 and V2 variants differ from each other by 3 nucleotides (bp) and their encoding proteins differ by one amino acid (aa) (Fig. 1A); the V3 variant is missing the in-frame coding exon 4 compared to the V1 transcript, resulting in an isoform lacking an internal 48 bp (16 aa, Fig. 1A) exon 4 compared to V1. The V4 is the shortest transcript lacking three consecutive internal coding exons compared to V1. These splicing defects result in translation initiation from an alternate start site and result in a gene that encodes an HMMR protein with a unique N-terminus compared to the V1 transcript (Fig. 1A). On the Fig. 1B, the HMMR-R3 peptide sequence used in vaccine therapy is highlighted in gray.

Supplementary Fig 2. Single cell sorting strategy

Patient samples were processed using Ficoll gradient. Next, samples were stained for PC and myeloid cell surface markers, and CD138+ PC and CD33+ myeloid cells were sorted into the 96 well-plates.

Supplementary Fig 3. Impact of SNVs on HMMR splicing

HEXplorer score profiles of HMMR exon 4 and flanking introns 3 and 4. The HMMR nucleotides are plotted along the x-axes, while y-axes display HEXplorer score values (HZEI). HEXplorer score differences between mutant and wile-type HMMR segments show the effects of the GVs on splice site usage and predict potential splicing regulatory elements. The blue bars represent the HZEI scores of the wild-type HMMR sequences, while black bars represent the HZEI scores of mutated sequences. On the figure, HEXplorer profiles of wild-type HMMR sequences are displayed as blue bars, while HEXplorer profiles of mutated sequences are shown as black bars. AG splice acceptor dinucleotides are marked by the red bars, while GT dinucleotides are marked by the yellow bars; numbers under the bars represent splice site strength defined as H-bond scores (HBS) or MaxEnt scores. Figure (A) shows HEXplorer score profile of the HMMR segments with four SNPs, while Figure B, C, D, and E display HEXplorer score profiles of the HMMR segments with SNPs rs561052191 (T>G), rs1235517851 (A>G), rs1175449655 (G>A) and rs767100503 (T>C), respectively. Locations of the plotted regions of the HMMR exon 4 and introns 3 and 4 are schematically shown on each HEXplorer profile.

Supplementary Figures 4 Evaluation ASO delivery efficiency in MM cell line

Representative flow cytometry result. The ASOs targeting HMMR FL and variants were delivered by gymnosis. The ASOs were tagged with GFP. In Fig gray-filled peaks represents negative control, while blue and green-shaded peaks represent samples expressing ASOs.

Supplementary Fig 5. in silico analysis of the impact of HMMR mutations on binding affinity of splicing factors: high score ESE motifs in the MT HMMR intron 3 and exon 4

Bar graphs represent scores above the threshold for the ESE motifs within the wild-type and mutant haplotypes. Arrows indicate the signal for SRp40 motif disappears when the mutation (G>A) is introduced into the wild-type sequence.
